# Maternity Homes: The Henry Saxon Snell Prize

**Published:** 1922-08

**Authors:** A. Saxon Snell

**Affiliations:** 9 Bentinck Street, Manchester Square, W.1.


### CORRESPONDENCE.

[Correspondence on all subjects is invited, but we cannot in any way be responsible for the opinions expressed by our correspondents, who must give name and address as a guarantee of good faith, but not necessarily for publication. Correspondents are reminded that conciseness greatly facilitates early insertion.]

### Maternity Homes: The Henry Saxon Snell Prize.

*To the Editor of* THE HOSPITAL AND HEALTH REVIEW.

SIR,—I desire to draw the attention of your readers to the subject set for this prize for the current year, viz., " A Maternity Home and Infant Welfare Centre." In the cause of social progress, these institutions are bound to have an importance in the future which is as yet little realised. Although a comparatively small number of specially designed buildings have been erected (and these are, to some extent, tentative), most Health Authorities have established Homes, if only in converted premises. In due course, properly designed buildings will be required all over the country, and those architects who have made a study of the subject may well hope to reap the benefit. In this connection, the prize offers a good opportunity for at least the commencement of such a study, and it is hoped that many practising architects will enter as competitors. Indeed, this is much to be desired. A memorandum setting forth in detail the requirements and also some information of buildings and writings for reference has been prepared by me and approved by the Council of the Royal Institute of British Architects. A copy of this will be sent to each competitor. The monetary value of the prize has been raised this year to £60.

Yours faithfully,
A. SAXON SNELL.

9 Bentinck Street,
Manchester Square, W.1.

### A Central Fund for Provincial Hospitals.

*To the Editor of* THE HOSPITAL AND HEALTH REVIEW.

